# A Fault Diagnosis Method of Modular Analog Circuit Based on SVDD and D–S Evidence Theory

**DOI:** 10.3390/s21206889

**Published:** 2021-10-18

**Authors:** Peng Sun, Zhiming Yang, Yueming Jiang, Shaohua Jia, Xiyuan Peng

**Affiliations:** School of Electronics and Information Engineering, Harbin Institute of Technology, 150100 Harbin, China; 15B301006@hit.edu.cn (P.S.); 14B901009@hit.edu.cn (Y.J.); 17S001076@hit.edu.cn (S.J.); pxy@hit.edu.cn (X.P.)

**Keywords:** SVDD, D–S evidence theory, state detection, fault diagnosis model

## Abstract

In the actual fault diagnosis process of an analog circuit, there is often a problem due to the lack of fault samples, leading to the low-accuracy of diagnostic models. Therefore, using positive samples that are easy to obtain to establish diagnostic models became a research hotspot in the field of analog circuit fault diagnosis. This paper proposes a method based on Support Vector Data Description (SVDD) and Dempster–Shafer evidence theory (D–S evidence theory) for fault diagnosis of modular analog circuit. Firstly, the principle of circuit module partition is proposed to divide the analog circuit under test, and the output port of each module is selected as test point. Secondly, the paper extracts the feature of the time-domain and frequency-domain output signals of the circuit module through Principal Component Analysis (PCA). Thirdly, four state detection models based on SVDD are established to judge the working state of each circuit module, including TSG, TSP, FSG, and FSP state detection model. Finally, the D–S theory is introduced to integrate the test results of each model for locating fault circuit module. To verify the effectiveness of the proposed method, the dual bandpass filter circuit is selected for simulation and hardware experiment. The results show that the proposed method can locate the analog fault effectively and has a higher diagnosis accuracy.

## 1. Introduction

With the rapid development of the electronics industry, the integration and complexity of electronic devices significantly improved. The emergence of various electronic products brought great convenience to people’s daily lives and plays a vital role in the industrial production. Electronic systems are widely used in the field of aerospace, transportation, communication, and so on. The occurrence of electronic system failures directly affects national security and people’s lives. Therefore, it is of vital importance to improve the reliability of electronic systems.

Fault diagnosis is one of the effective means to improve the reliability of electronic systems. The definition of fault diagnosis is that engineers use various detection methods and test methods to detect the state of the system or equipment (normal operation or fault) and determine the location of the faults. Timely and accurate fault diagnosis is conducive to ensuring stable operation of the system and reducing the risk of major failures effectively. Precise locating fault can reduce the cost and workload of maintaining circuit systems, and significantly improve the reliability of the electronic system.

Theoretical analysis and practical applications show that analog circuits are more prone to failure than digital circuits. Although the proportion of analog circuits in electronic system does not exceed 20%, more than 80% of faults come from analog circuits [1]. With the development of semiconductor technology and integrated circuits, more than 60% of integrated circuits are currently a hybrid circuit composed of digital circuits and analog circuits. Although the analog circuit portion only accounts for 5% of the circuit area, the test cost accounts for 95% of the total test cost [2]. The value of analog circuits’ input and output signals and the component parameters are continuous; meanwhile, there are inevitable tolerance and nonlinear components in the analog circuits, the analog fault types including hard faults, soft faults, and intermittent faults are various [3,4,5]. Therefore, these factors increase the complexity of analog circuit fault diagnosis.

Analog circuit fault diagnosis problem is considered to be a classification problem, and researchers are actively exploring ways to develop many effective fault diagnosis methods for analog circuits. For example, experts applied the intelligence technology including expert system methods, fuzzy theory, artificial neural network, and support vector machines to build the multiclassification models to diagnose the analog faults [6,7,8]. Song et al. used the RMS specification to measure the sensitivity of the output response of the circuit under test obtained by multiresolution analysis and used the neural network as a classifier for fault diagnosis [9]. Sheikhan M et al. used the particle swarm optimization algorithm to determine the number of nodes in the hidden layer of the neural network module, and used the training modules of output-implicit layer weight optimization to achieve the multicircuit diagnosis [3]. Long et al. selected the optimal fault eigenvector to detect the potential fault of the analog circuit, through the least squares support vector machine method and the particle swarm optimization method based on Mahalanobis distance [10]. Han et al. used the particle swarm optimization algorithm to achieve the quantitative diagnosis of faults in analog circuits under tolerance conditions when they established and solved linear programming equations for node voltage sensitivity and node voltage increment [11]. Yang et al. presented a systematic method based on a neural network that utilizes a genetic algorithm (GNN) and the deviation space to diagnose faulty behavior in analog circuits under test (CUTs) [12]. Ying et al. developed a neural-network-based fault diagnosis approach of analog circuits using maximal class separability-based kernel principal components analysis (MCSKPCA) as preprocessor. This approach can detect and diagnose faulty components efficiently in the analog circuits by analyzing their time responses [13]. Piotr Bilski et al. presented an automated method for analog system diagnostics using Wavelet Transform and fuzzy logic methods, which aim to detect and localize multiple faults in noisy conditions [14]. Jing et al. developed a completely automatic classification system that estimate the best value of the SVM classifier parameters and select optimization feature subspaces in analog circuit fault diagnosis through a hybrid particle swarm optimization algorithm framework [15]. Zhang et al. used deep confidence network optimized by quantum-behaved particle swarm optimization (QPSO) algorithm to extract circuit features for analog circuit incipient fault diagnosis [16].

Although many research institutes and scholars completed quite a lot of research work in the field of fault diagnosis, the following problems still exist in the multiclassification fault diagnosis methods in practical engineering. Firstly, the multiclassification method determines the circuit state as normal or fault by collecting the output signals of each test point. It is necessary to obtain enough normal state samples (positive samples) and fault state samples (negative samples) when training the classifier. However, the fault states of electronic equipment are numerous and difficult to estimate, and all fault states of the circuit cannot be found in actual engineering projects. Therefore, it is impossible to establish multiple classifiers that can detect all faults of the circuit. In addition, multiclassification models only can separate them very well when different fault classes are isolated enough. This requires the engineers to understand the working characteristics of the circuit well before considering the types of faults. Misclassification and unclear definition lead to the difficulties in training process and higher rates of misclassification.

To solve the above problems, this paper proposes a one-classification state diagnosis method for modular analog circuit. The circuit features are extracted by principal component analysis (PCA) from the signals which are acquired from test points. To reduce the effect of the insufficient fault samples scales on the diagnosis accuracy, the classifier is built by the Support Vector Data Description (SVDD), which only needs the normal samples to train the model. For improving higher diagnosis accuracy, the paper trains four SVDD state detection models, and the fault detection accuracy of these models are easily obtained. Then, these accuracy are used to construct the evidence bodies of Dempster–Shafer evidence theory (D–S evidence theory) and a fault diagnosis model based on SVDD and D–S theory is established. D–S evidence theory is used to fuse the fault detection accuracy of four state detection models to construct D–S evidence bodies. Four SVDD state detection models include time-domain SVDD state detection model with Gaussian kernel (TSG detection model), frequency-domain SVDD state detection model with Gaussian kernel (FSG detection model), time-domain SVDD state detection model with polynomial kernel (TSP detection model), and frequency-domain SVDD state detection model with polynomial kernel (FSP detection model). The D–S evidence theory can be used to set the confidence level for the fault diagnosis results of different state detection models, and the location of the fault module can be determined by combining the actual measurement data with D–S evidence bodies.

The method proposed in this paper has the following advantages: in the case of no negative class samples (fault state samples), the fault diagnosis model can be established only by using positive samples. The method has strong processing ability for small sample, high-dimensional, and noisy data, and has a good classification effect. Using the D–S evidence theory can synthesize test results of multiple state detection models and reduce the impact of the single model’s misjudgment on the final results effectively. It can significantly improve the accuracy of fault location.

The rest of this paper is organized as follows: Section 2 introduces the preliminaries including PCA, SVDD and D–S evidence theory. The proposed fault diagnosis method is further introduced in Section 3 with a case study. The simulation and hardware experiment are described in Section 4 to verify the validity of the proposed method. Finally, paper is concluded in Section 5.

## 2. Preliminaries

### 2.1. Principle Component Analysis

Principal component analysis (PCA) originated from statistics, and its concept was proposed by Pearson and improved by J.E. Jackson and Hotelling [17]. The essence of PCA is data replacement. A small number of variables can obtain raw data information and replace it for subsequent processing. The replaced data is a linear combination of the original data. Using this method for feature extraction can compress the amount of data, simplify the data features, and reduce dimensionality [18,19].

The purpose of PCA is to decorrelate. The problem contradiction is more prominent through coordinate transformation. According to the analysis of 2D space, the dispersion degree of data can be expressed by variance. The larger the variance of the data in a certain direction, the more information they cover. Through the coordinate transformation, the original data are transformed into a set of new data that are irrelevant. Among them, the linear combination with the largest variance is the first principal component, and the principal components are obtained in turn and are not related to each other. The steps for feature extraction using PCA are as follows:

#### 2.1.1. Data Standardization Processing

It is assumed that the obtained raw data **X** is data of *n* rows and *p* columns. *n* represents the number of samples and *p* represents the dimension of the data. Through the standardization of data processing, the influence of different dimensions within the data is removed, and the amount of calculation in the later stage is reduced. The data after standardization data is represented by **Y**.

(1) $$Y=\left[X-1_{n}u^{T}\right]D_{\sigma }^{-1/2}$$

Among them, $1_{n}$ represents an *n*-dimensional column vector where all elements are 1, $D_{\sigma }=diag\left(\sigma_{1}^{2},\sigma_{2}^{2},\ldots ,\sigma_{p}^{2}\right)$ is a variance matrix, and $u={\left[u_{1},u_{2},\ldots ,u_{p}\right]}^{T}$ is a mean vector matrix.

#### 2.1.2. Establishing a Correlation Matrix

We construct a matrix $\sum =Y^{T}Y/\left(n-1\right)$, the eigenvalues of ∑ can be obtained and arranged as $\mu_{1},\mu_{2},\ldots ,\mu_{p}$ from large to small. Then, the corresponding orthogonal normalized eigenvectors $V_{1},V_{2},\ldots ,V_{p}$ are obtained. $V_{1}$ represents the direction in which the data set Y changes the most. Therefore, each component of $V_{1}$ is the first principal component as a linear combination of the obtained variables. Similarly, with $V_{2}$ as the coefficient, the linear combination is the second principal component.

#### 2.1.3. Confirming the Number of Principals

The variance contribution rate $a_{i}$ is introduced here to indicate the importance of the principal.

(2) $$a_{i}=\frac{\mu_{i}}{\sum_{i=1}^{p}\mu_{i}}$$

We define the cumulative contribution of the variance as $a^{m}=\sum_{i=1}^{m}a_{i}$ and set an appropriate threshold for it to determine the number of principals that can replace all sample information. The minimum *m* value that makes the cumulative contribution contribution rate greater than the threshold is the number of primary elements.

#### 2.1.4. Calculate the Principal Component Value and Create a New Sample

**Figure 1:**
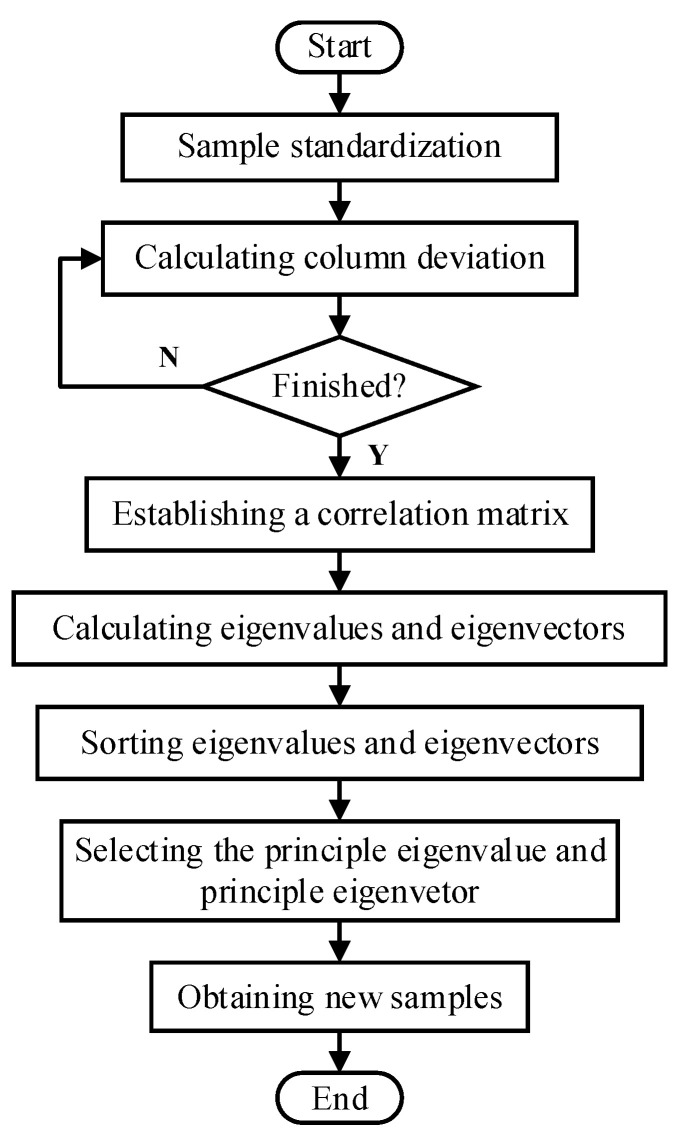
Algorithm flow chart of PCA.

The PCA algorithm flow is shown in Figure 1. The feature extraction is performed by PCA, and the data compression is effectively completed. PCA eliminates the redundant information and reduces computational complexity. The obtained data retains most of the original data, so as to facilitate efficient diagnosis in the later stage. To obtain a better description of the data, the selected principal element cumulative contribution rate threshold is 99% in the later diagnosis process.

### 2.2. Support Vector Data Description

Support vector data description (SVDD) is based on statistical learning theory and inherits its advantages [20]. The core idea of SVDD is to find a hypersphere with the smallest radius that can contain all training sample data by mapping the sample data to a high-dimensional space. The boundary of the hypersphere is used as the basis for classifying similar and heterogeneous.

SVDD is one-class classification, which is trained by using one-class samples (positive samples). To a certain degree, it solves the problem of sample missing. Especially in the field of fault diagnosis, it has a certain practical significance for the case that all the fault samples cannot be obtained. The output signal of the circuit module under normal condition is used as positive sample for training. If the measured signal data are distributed inside the hypersphere, the output is in normal state. On the contrary, the output is in fault state.

SVDD establishes a closed compact hypersphere with a center a and radius *R*. The hypersphere contains as many samples as possible. The relaxation factor is added to increase its robustness. The supersphere is satisfied:

(3) $$\begin{array}{cc} \; & \min\;F\left(R,a\right)=R^{2}+C\sum_{i=1}^{N}\xi_{i}\\ \; & s.t.\;||\phi \left(x_{i}\right)-a{\left|\right|}^{2}\leq R^{2}+\xi_{i},\;\xi_{i}\geq 0 \end{array}$$

Among them, $\xi_{i}$ is the relaxation variable, and *C* is the penalty factor, which is used to evaluate the relationship between the volume of the hypersphere and the sample deviation.

To judge whether the test sample *z* falls on the inner-side or the outer-side of the hypersphere, it is only necessary to compare the relationship between the distance *e* from the center of the sphere and the radius *R*. The expression of the supersphere radius *R* is:

(4) $$\begin{array}{cc} R^{2}= & ||\phi \left(x_{*}\right)-a{\left|\right|}^{2}=<\phi \left(x^{*}\right)\cdot \phi \left(x^{*}\right)>-\\ & 2\sum_{i=1}^{N}\alpha_{i}<\phi \left(x^{*}\right)\cdot \phi \left(x_{i}\right)>+\sum_{i=1}^{N}\sum_{j=1}^{N}\alpha_{i}\alpha_{j}<\phi \left(x_{i}\right)\cdot \phi \left(x_{j}\right)> \end{array}$$

The equation for *z* to the center of the sphere α is:

(5) $$\begin{array}{cc} D^{2}= & <\phi \left(z\right)\cdot \phi \left(z\right)>-2\sum_{i=1}^{N}\alpha_{i}<\phi \left(z\right)\cdot \phi \left(x_{i}\right)>\\ \; & +\sum_{i=1}^{N}\sum_{j=1}^{N}\alpha_{i}\alpha_{j}<\phi \left(x_{i}\right)\cdot \phi \left(x_{j}\right)> \end{array}$$

In the above formula, we need to calculate the inner product of samples in high-dimensional space, which leads to huge computational load and sometimes even impossible to calculate. To solve the problem, we can replace the nonlinear function by introducing a kernel function

(6) $$K\left(x_{i},x_{j}\right)=<\phi \left(x_{i}\right)\cdot \phi \left(x_{j}\right)>$$

The model is improved by modifying the parameters of the kernel function to adjust the classification effects. Gauss kernels and polynomial kernels are two common kernel functions.

- Gaussian kernel function

(7) $$K\left(x,y\right)=exp\left(-\frac{||x-{y||}^{2}}{2\sigma^{2}}\right)\;\sigma >0$$

- Polynomial kernel function

(8) $$K\left(x,y\right)={\left[\left(x\cdot y\right)+1\right]}^{d}\;d\in N$$

When the state detection model is established by using the SVDD Gaussian kernel and the polynomial kernel, the detection rate depends heavily on the parameters of the kernel function. Since the parameters σ and *C* of the Gaussian kernel and the polynomial kernel parameters *d* and *C* have certain influence on the established hypersphere boundary, it is necessary to find a suitable and effective method to optimize the parameters. The parameter selection methods mainly include grid search, gradient descent method, and particle swarm algorithm. In this paper, the method of grid search and cross-validation are used to optimize the parameters.

### 2.3. D–S Evidence Theory

D–S evidence theory, developed and perfected by Dempster and Shafer, is based on a mathematical uncertainty reasoning method [21,22,23,24]. A problem is described by multiple evidences in D–S evidence theory. These evidences are integrated according to certain rules to make final decisions. This fusion method can remove some contradictory and redundant information. To integrate multiple evidences, D–S evidence theory needs to construct evidence body for each event, and give fusion rules. Finally, the fusion evidence is used as decision-making basis. The following is the process of evidence fusion.

The ultimate goal of D–S evidence theory is to complete the identification. So a nonempty set θ is introduced as the recognition framework, which contains several kinds of events. Each of events is independent and in a repulsive relationship. A basic probability (BPA) is assigned to various events and represented by the *m* function. $m:2^{\theta }\overset{}{\to }\left[0,1\right]$ and it satisfies:

(9) m(Φ)=0

(10) ∑m(X)=1

The *m* function assigns a probability to each event, which represents the level of trust in the event. It indicates the extent to which this evidence supports the incident, and m() indicates the uncertainty of the evidence. To describe the degree of trust in an event better, a confidence interval is introduced, represented by the trust function (Bel) and the likelihood function (Pl):

(11) $$Bel\left(A\right)=\sum_{B\subseteq A}m\left(B\right)$$

(12) $$Pl\left(A\right)=\sum_{B\cap A\neq \phi }m\left(B\right)$$

**Figure 2:**
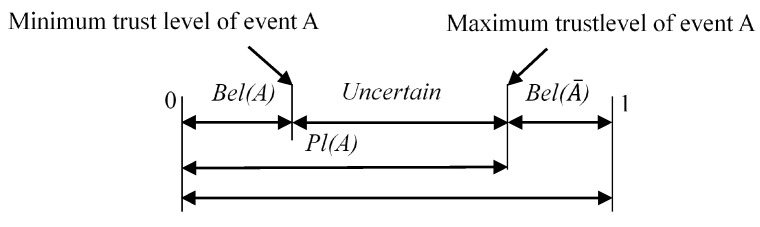
Trust interval representation graph.

The trust function Bel(*A*) represents the support degree for event A, and the likelihood function Pl(*A*) represents the degree of nonrejection to event A, that is, there is some uncertainty degree. Their relationship is shown in Figure 2.

The use of D–S evidence theory for fusion is achieved by the different levels of trust in the same event. $m_{j}\left(A_{i}\right)$ indicates that event $A_{i}$ gets the trust function of evidence *i*.

(13) $$M_{final}\left(A\right)=\frac{\sum_{B\cap A_{i}=A}\prod_{j=1}^{n}m_{j}\left(A_{i}\right)}{1-\sum_{B\cap A_{i}=\phi }\prod_{j=1}^{n}m_{j}\left(A_{i}\right)}$$

The fusion results of multiple evidences are independent of the order of fusion. $M_{final}\left(A\right)$ indicates the final evidence after fusion. The recognize result of new evidence can be obtained according to the Decision Principle. In the final evidence $M_{final}$, $M_{final}\left(Max\right)$ is the maximum value. $M_{final}\left(Max\right)$ is at least $\lambda_{1}$ greater than the other values. At the same time, $M_{final}\left(Max\right)$ minus $M_{final}\left(uncertain\right)$ is greater than $\lambda_{2}$ and $M_{final}\left(uncertain\right)$ should be less than $\lambda_{3}$. Thereinto, $\lambda_{1}$, $\lambda_{2}$ and $\lambda_{3}$ mean three decision thresholds in D–S decision theory, they are used to constraint the value of $M_{final}$ to be optimal and reasonable. In the actual applications, these three values will be artificially set.

## 3. Establishment of Fault Diagnosis Model

**Figure 3:**
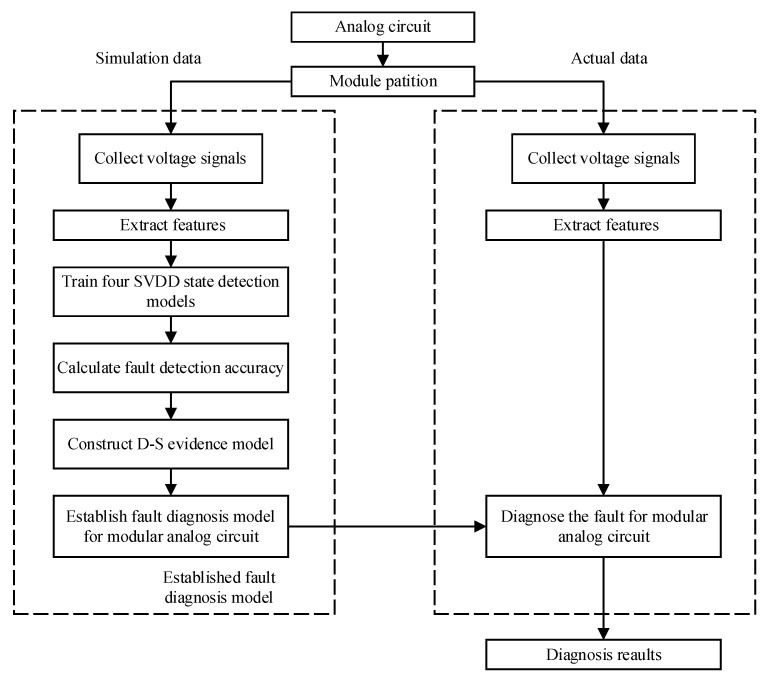
A proposed fault diagnosis method based on SVDD and D–S evidence theory.

For a given actual circuit, the structure is divided into modules according to functions, as shown in Figure 3. The following is a case study to further explain the proposed scheme.

Firstly, the circuit under test (CUT) is divided into several interconnected circuit modules according to different functions. The modules have relatively independent structures and functions, and the output ports of each module is selected as test point. The above description is the division principle used in this paper. Secondly, with the help of circuit simulation technology, the principal component analysis (PCA) method is used to extract the features of the sampled data which are collected from time-domain and frequency-domain output signals in the circuit fault-free state. Thirdly, the SVDD method is used to train the state detection models which are established only by positive samples. It is easy to obtain fault detection accuracy of these four state detection models for different circuit modules. Finally, evidence bodies in D–S evidence theory are generated by using the fault detection accuracy of the four models. The fault diagnosis method combining the state detection models based on SVDD with the fusion ability of D–S evidence theory is called fault diagnosis model. This trained model can be applied to the fault diagnosis process of the actual circuit. When the measured data of the circuit are sent to this diagnosis model, it can judge the working state of the circuit and locate the fault module with high accuracy.

### 3.1. Extracting Features

**Figure 4:**
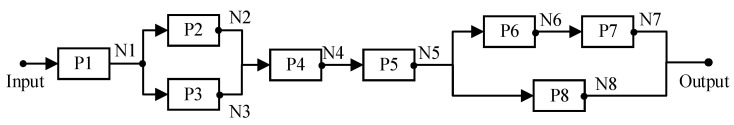
Structure of Circuit Modules.

Taking the circuit in Figure 4 as an example.The circuit is divided into 8 modules, which are named from P1 to P8. The output ports corresponding to each module are marked N1 to N8. These nodes are used as the test points for collecting signals in the time domain and frequency domain.

To obtain the simulation data, the pulse signal is selected as the power supply excitation signal when collecting the time-domain voltage signal and the frequency sweep signal in the simulation process. Then, the PCA method is used to extract the features of the positive samples, so as to reduce the operational dimension and improve the computing speed on the premise of maintaining the maximum amount of information. For the circuit structure shown in Figure 4, node N1–N8 are selected as the test points. Firstly, circuit simulation technology is used to obtain positive samples under normal working conditions. The pulse signal or sweep signal is taken as the test excitation of the analog circuit, and the voltage signals of circuit modules are collected. Since we can only get a set of data in simulation when the circuit parameters are fixed, we set a tolerance for each component, and carry out a certain number of Monte Carlo analysis. The simulation data collected from each test point are taken as the data of the corresponding circuit module in fault-free state.

Since the redundancy of information between test points and the excessive sample dimension, the complexity and convergence time of training the SVDD model will be greatly increased. Therefore, PCA is used to extract features from the data. The threshold of cumulative contribution rate of principal component in PCA method is set to 99%. The samples are normalized to eliminate the dynamic deviation among the dimensional features. Finally, the obtained samples are used to train the state detection model based on SVDD.

### 3.2. Obtaining the Fault Detection Accuracy of the State Detection Models

We use positive samples after data processing to train SVDD single classifier so that it can distinguish whether the input data belongs to fault-free signal or fault signal. In other words, when the input data of the classifier is a fault-free sample, the output value of the classifier is 1; when the input data is the fault sample, the classifier output is 0. The output value of the model also indicates whether the circuit module is working normally or not.

**Figure 5:**
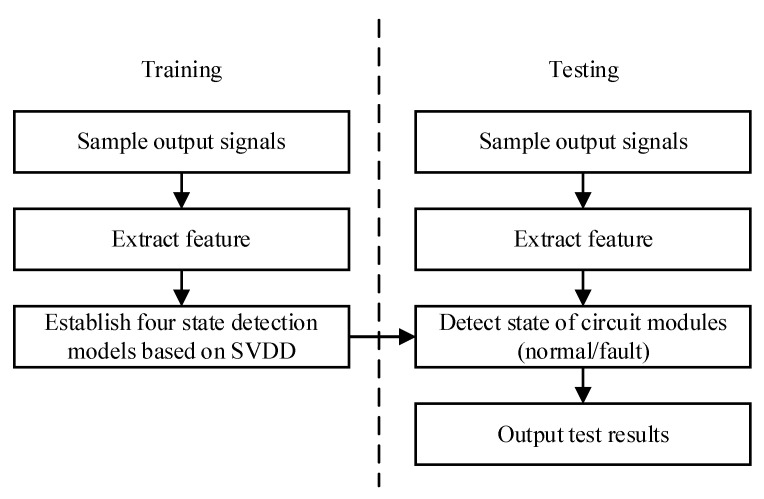
Flow chart of state detection models.

First of all, because a circuit module contains many components, the average fault detection rate of the model is defined as the average probability that a state detection model correctly judges the working states of the circuit modules when different components fail. To obtain the fault detection rate for different circuit fault modules, the state detection models should be established for each circuit module. The purpose of establishing these models is to judge the state of each circuit module by the output signals. The performance of circuit faults is different in time domain and frequency domain, and the selection of different kernel functions in the training of SVDD models also affect the classification accuracy. Therefore, to make full use of the advantages of different state detection models, we choose Gaussian kernel and polynomial kernel as two kernel functions, and respectively use positive samples in time domain and frequency domain for training state detection models. So we have four state detection models: TSG model, TSP model, FSG model, and FSP model. The training methods of these four state detection models are the same, and different training samples and kernel functions are only needed. The process of model establishment is shown in Figure 5, which mainly includes the training process and the process of obtaining fault detection rate.

Training process: samples processed by PCA are used to train state detection models. Gaussian kernel function and polynomial kernel function are selected to establish the hypersphere describing the training samples. The SVDD state detection model corresponding to this module is established. The training process is to find a hypersphere that can contain all the training sample data with the smallest radius, and make it possible to distinguish the category of a sample, so as to complete the state detection. Other state detection models can be trained by changing the kernel function or domains.

Obtaining fault detection accuracy: we need to provide the same excitation signal to analog circuits as the training process, and use PCA method to extract the features of the data collected from each test point. The fault detection accuracy of the state detection models are tested with the processed data. To obtain enough fault samples used to calculate the fault detection accuracy, we set the parameter drift of 40% and 50% respectively for the fault component on the basis that each component contains 5% tolerance in the simulation software. When each component parameter has drifeted from 40% or 50% in one modular analog circuit, respectively. It demonstrates that this module is in the fault state. For the module P1–P8, the fault states will be decribed as P1 fault to P8 fault. The output signals of each test point obtained by Monte Carlo analysis are fault samples. The state detection model is used to recognized the fault samples of the same module. The proportion of correctly detected faults to the total number of samples is the fault detection rate.

After judging the working state of each circuit module, the position of fault module can be located by combining Simple Logic Discrimination Method (SLDM). In other words, due to the slave relationship between modules, when a module fails, it has no influence on the output of the superior module, but transfers fault feature to the subordinate module. In the direction of the circuit signal transmission, the first module whose state detection result is ‘abnormal’ is recognized as the fault module.

When the state detection result of node N4 is 1 (normal state) and the detection result of node N5 is 0 (abnormal state), we can judge that the fault module is the module P5 corresponding to node N5. However, the fault detection rate of the state detection model is not 100%. If the state of node 4 is wrongly judged as ‘abnormal’ (the output is 0) in the state detection, the fault module located by SLDM is P4 instead of the real fault module P5. Therefore, it is more demanding to use SLDM for fault location. It requires SVDD state detection model to have a very high detection rate for each module. Error detection of a single model leads to fault location errors, and the accuracy of location greatly depends on the state detection results of a single model. In addition, different state detection models have different fault detection accuracy for the same circuit module. It is impossible to give full play to the advantages of other models only based on a single model. Therefore, we introduce D–S evidence theory and construct 4 pieces of evidence bodies with fault detection accuracy of four state detection models. The purpose is to assign a certain weight or degree of credibility to the actual state detection results.

This method integrates the detection information of four state detection models to complete fault location. This fault diagnosis method based on SVDD state detection models and D–S evidence theory is called “fault diagnosis model”. This method has reliable fault diagnosis results and effectively reduces or avoids the influence of wrong state detection model results on fault location. For example, for the voltage signal collected from node N4, it is assumed that the result of the first state detection model is abnormal (output is 0), and the result of the other three state detection models is normal (output is 1). The ‘fault diagnosis model’ can reduce the confidence probability of the state detection result of model 1 and the contribution to the fusion judgment results, so that the final judgment result of module P4 corresponding to node N4 is ‘normal’. This method increases the tolerance of misclassification results and improves the accuracy of fusion detection results.

### 3.3. Constructing D–S Evidence Theory Body

**Figure 6:**
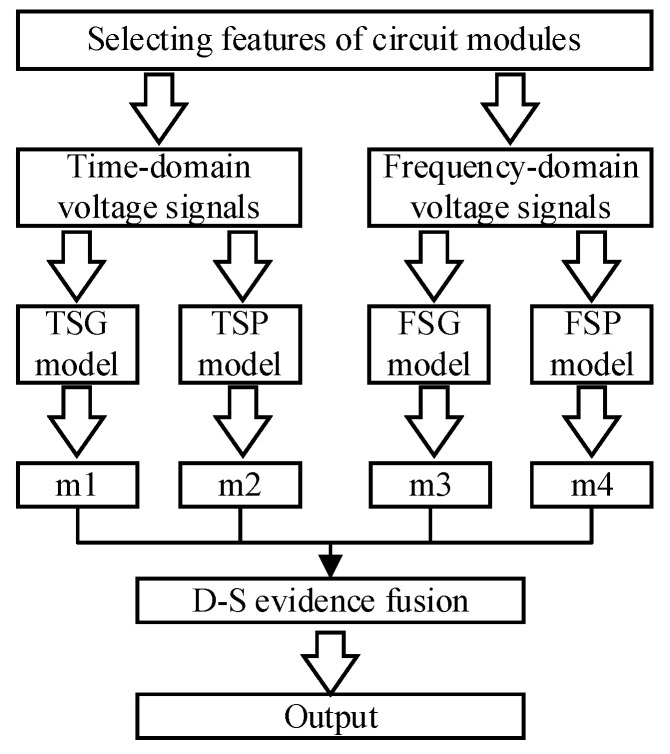
Relationship between D–S evidence bodies and state detection models.

The D–S evidence bodies are calculated by using the fault detection accuracy of the four state detection models obtained in Section 3.2. D–S evidence bodies $m_{1}$, $m_{2}$, $m_{3}$ and $m_{4}$ correspond to TSG, TSP, FSG, and FSP state detection models respectively. The recognition framework of D–S evidence fusion includes P1 fault to P8 fault, normal state and uncertain state. The relationship between the evidence body and the state detection model, as well as the process of circuit module fault diagnosis are shown in Figure 6.

**Figure 7:**
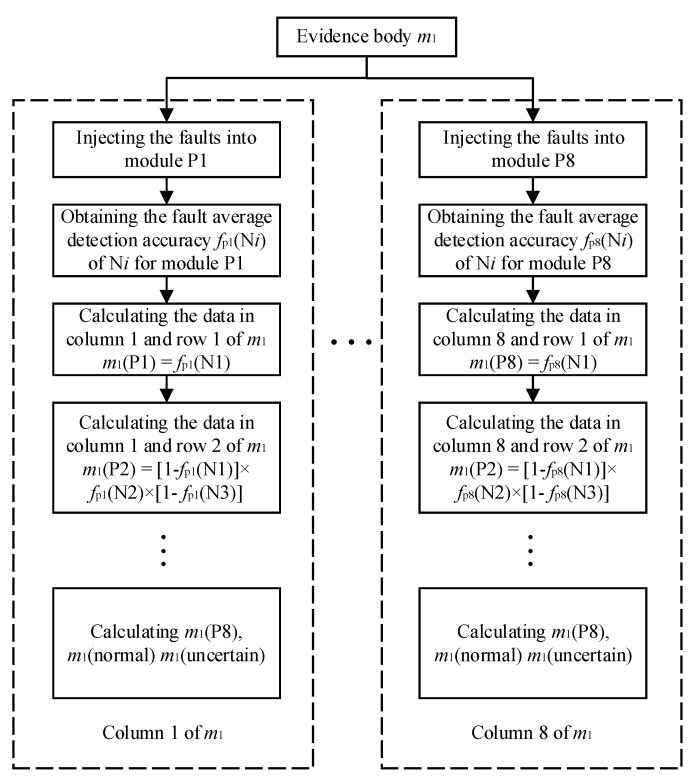
Construction process of evidence body $m_{1}$.

The basis of evidence fusion is the distribution of confidence probability for state detection results through D–S evidence. For example, the evidence body of the TSG state detection model is $m_{1}$, and each column of evidence body $m_{1}$ covers the support degree of the state detection results to each module fault and the normal state. SLDM and fault detection accuracy of state detection models are used to construct evidence body. The construction process is shown as Figure 7, and the detailed steps of $m_{1}$ are also as follows.

Step 1: column *i* of D–S evidence body $m_{1}$ indicates the support degree of TSG state detection model results for module P*i* fault. Here, we assume that module P1 fails. The symbol $f_{pi}$ (N*i*) is used to express the fault detection rate of TSG model at the node N*i* when the module P*i* fails. The fault average detection rate of the node N1 is expressed as the probability of the model detection of abnormality at N1. It indicates the support degree of the TSG state detection for module P1 failure, recorded as $m_{1}$(P1), $m_{1}$(P1) = $f_{p1}$ (N1). This value is the data in column 1 and row 1 of the evidence body $m_{1}$.

Step 2: calculate the support degree of the TSG state detection for module P2 failure. It is equal to the probability that the node N1 detection result is normal, the node N2 result is abnormal, and the node N3 result is normal. $m_{1}$ (P2) = [$1-f_{p1}$ (N1)] $\times f_{p1}$ (N2) × [$1-f_{p1}$(N3)]. This value is the data in column 1 and row 2 of $m_{1}$.

Step 3: calculate the support degree of the TSG state detection for module P3 failure. It is equal to the probability that the node N1 detection result is normal, the node N2 result is normal, and the node N3 result is abnormal. $m_{1}$ (P3) = [$1-f_{p1}$ (N1)] × [$1-f_{p1}$(N2)] $\times f_{p1}$(N3). This value is the data in column 1 and row 3 of $m_{1}$.

Step 4: calculate the support degree of the TSG state detection for module P4 failure. It is equal to the probability that the nodes N1, N2 and N3 detection results are normal, the node N4 result is abnormal. $m_{1}$ (P4 ) = [$1-f_{p1}$ (N1)] × [$1-f_{p1}$(N2)] × [$1-f_{p1}$(N3)] $\times f_{p1}$(N4). This value is the data in column 1 and row 4 of $m_{1}$.

Step 5: by analogy, $m_{1}$(P5) , $m_{1}$ (P6), $m_{1}$ (P7), $m_{1}$ (P8), and $m_{1}$ (normal) are calculated, respectively. $m_{1}$ (normal) is equal to the probability that all nodes are normal.

Step 6: because we inject a single fault in simulation, $m_{1}$ (uncertain) is defined as the probability that N2 and N3 detection results are both abnormal, other results are normal.

When using the evidence body, a column of the evidence body is selected according to the result of state detection model, and the final evidence $m_{final}$ is fused by Formula (13). To locate faults according to $m_{final}$, we need to abide by following three principles, which are called Fault Module Decision Principle (FMD principle). In the final evidence $m_{f}inal$, *m*(A) is the maximum, and *m*(A) is at least $\lambda_{1}$ greater than the other values. At the same time, *m*(A) minus *m* (uncertain) is greater than $\lambda_{2}$ and *m*(uncertain) should be less than $\lambda_{3}$. If FMD principle is met, circuit module A is considered a fault module.

## 4. Experiment and Results

### 4.1. Establishing Fault Diagnosis Model for the Dual Band-Pass Filters

**Figure 8:**
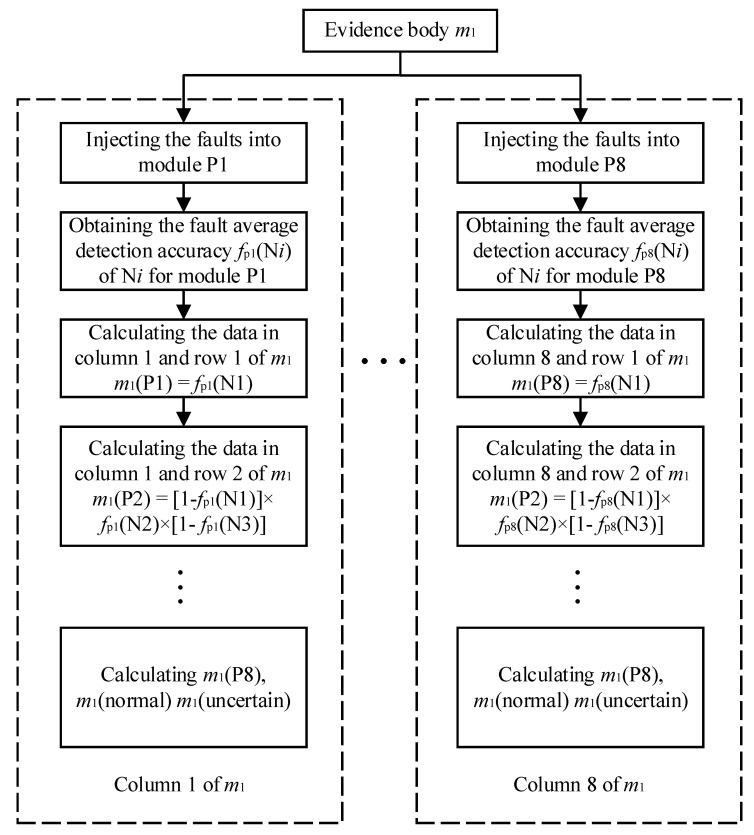
Schematic diagram of dual band-pass filter circuit.

To prove the effectiveness of the proposed circuit fault diagnosis method based on SVDD and D–S evidence theory, a dual bandpass filter circuit is selected for experiments. The circuit is composed of a Sallen–Key circuit, and the circuit structure is as shown in Figure 8.

At the beginning of the experiment, we need to establish a fault diagnosis model for the circuit through simulation experiments. According to the division principle, the circuit is divided into five modules P1–P5, and the components and functions are shown in Table 1. Modules P2 and P3 are parallel modules, and they are in series with other modules. The output nodes of the five modules are selected as five test points. The passbands of the dual bandpass circuit are 50 Hz–1 kHz and 5 k–50 kHz, respectively.

In PSPICE, a pulse signal with a period of 500 μs, a pulse width of 20 μs, and an amplitude of 5 V is selected as the test excitation signal. 5% tolerance is set for all components. At first, the TSG state detection model is used to judge working state of module P1, and its detection accuracy are obtain as follows: 550 times of Monte Carlo analysis are carried out to obtain the time-domain voltage signal samples from node N1. PCA method is used to extract and normalize the sampled data. These 550 sets of sample data are the positive samples of the circuit module P1 under normal working conditions. 500 sets of samples are used to train SVDD model, and the remaining 50 samples are used to calculate the accuracy of the model. For each component in module P1, 40% and 50% parameter drifts are respectively injected into the components as fault states. 300 Monte Carlo analyses (50 sets for each fault module) are performed separately, and the obtained fault samples will extracted by PCA method are used as the testing data of the SVDD model. Based on TSG state detection model, the fault detection accuracy from test point N1 to N5 are shown in Table 2 row 3. When different modules are in the fault states, respectively. The results are shown as Table 2.

The table shows that the fault detection effect of the TSG state detection model is ideal. When the component has 50% fault drift, its average detection rate is above 90%. However, the detection accuracy of the TSG state detection model for a few circuit fault modules needs to be improved. The FSG and FSP state detection models are established using the frequency-domain features of the circuit. Therefore, a sweep signal with an amplitude of 4 V and a frequency of 1 Hz–1 MHz is selected as the test excitation. The frequency-domain voltage signals acquired from the test points are used as positive samples. The method of establishing the FSG and FSP state detection models is similar, and are not described in this paper.

The detection accuracy at 40% and 50% failures in Table 2 are averaged as the average detection rate of the TSG model, as shown in Table 3. The D–S evidence body $m_{1}$ is established according to Table 3.

Step 1: when constructing the first column of data for the evidence body $m_{1}$, we assume that module P1 is faulty. According to the fault detection result of Table 3, the fault average detection rate of the node N1 is expressed as the probability of the model detection of abnormality at N1. It indicates the support degree of the TSG state detection for module P1 failure, recorded as $m_{1}$ (P1) = 0.9792. This value is the data in column 1 and row 1 of the evidence body $m_{1}$.

Step 2: calculate the support degree of the TSG state detection for module P2 failure. It is equal to the probability that the node N1 detection result is normal, the node N2 result is abnormal, and the node N3 result is normal. $m_{1}$ (P2) = (1 − 0.9792) × 0.7511 × (1 − 0.3303) = 0.0105. It is the data in column 1 and row 2 of $m_{1}$.

Step 3: calculate the support degree of the TSG state detection for module P3 failure. It is equal to the probability that the node N1 detection result is normal, the node N2 result is normal, and the node N3 result is abnormal. $m_{1}$ (P3) = (1 − 0.9792) × (1 − 0.7511) × 0.3303 = 0.0017. It is the data in column 1 and row 3 of $m_{1}$.

Step 4: calculate the support degree of the TSG state detection for module P4 failure. It is equal to the probability that the nodes N1, N2 and N3 detection results are normal, the node N4 result is abnormal. $m_{1}$(P4) = (1 − 0.9792) × (1 − 0.7511) × (1 − 0.3303) × 0.1952 = 0.0000. It is the data in column 1 and row 4 of $m_{1}$.

Step 5: calculate the support degree of the TSG state detection for module P5 failure. It is equal to the probability that the nodes N1, N2, N3 and N4 detection results are normal, the node N5 result is abnormal. $m_{1}$ (P5) = (1 − 0.9792) × (1 − 0.7511) × (1 − 0.3303) × (1 − 0.1952) × 0.1189 = 0.0000. It is the data in column 1 and row 5 of $m_{1}$.

Step 6: calculate the support degree of the TSG state detection for fault-free state. It is equal to the probability that the nodes N1, N2, N3, N4 and N5 detection results are all normal. $m_{1}$ (normal) = (1 − 0.9792) × (1 − 0.7511) × (1 − 0.3303) × (1 − 0.1952) × (1 − 0.1189) = 0.0025. It is the data in column 1 and row 6 of $m_{1}$.

Step 7: Calculate the support degree of the TSG state detection for uncertain state. It is equal to the probability that nodes N1, N4 and N5 detection results are normal, nodes N2 and N3 detection results are abnormal. $m_{1}$ (uncertain) = (1 − 0.9792) × 0.7511 × 0.3303 × (1 − 0.1952) × (1 − 0.1189) = 0.0052. It value is the data in column 1 and row 7 of $m_{1}$.

At this point, the first column of values in the evidence body $m_{1}$ was constructed, which is named as P1-fault evidence in $m_{1}$. The construction of other evidence is similar to the above process. The evidence bodies $m_{1}$, $m_{2}$, $m_{3}$ and $m_{4}$ are shown in Table 4, Table 5, Table 6 and Table 7.

In the actual detection process, four state detection models are used to judge the working state of each circuit module. The position of the fault circuit module is initially determined using the SLDM. It is easy to find the column corresponding to the fault in the evidence body. This column serves as a piece of evidence for the state detection model. Finally, the four pieces of evidences obtained are fused using Formula (13). The location of the fault module is determined by $m_{final}$.

The following is a detailed introduction to the use of evidence: if the fault location result of TSG model using SLDM is module P3, the third column in the evidence body $m_{1}$ will be taken as the first piece of evidence. Similarly, if the fault location result of TSP model is module P2, the second column in the evidence body $m_{2}$ will be taken as the second piece of evidence. According to the same method, if the fault location result of the FSG model is module P3, the third column in the evidence body $m_{3}$ will be taken as the third evidence. If the fault location result of the FSG model is normal state, the sixth column in the evidence body $m_{4}$ will be taken as the fourth evidence.

The result of the fusion using the D–S evidence theory is the last column of Table 8, which is calculated by Formula (13). Among them, 0.6960 is the maximum, which is far more than the second largest number of 0.1668. At the same time, $m_{final}$ meets FMD principle mentioned in Section 3.3. It indicates that circuit module P3 is fault. Although the fault location results of the four state detection models are not the same, the final diagnosis result is still module P3, which is consistent with our assumptions. Therefore, D–S evidence theory increases the error-tolerant rate of the single detection model, making the final diagnosis more reliable.

We established the fault diagnosis model of dual bandpass filter circuit. Next, we will test the accuracy of the fault diagnosis model through simulation and hardware experiment.

### 4.2. Simulation Experiment

Three hundred sets of failure samples and 200 sets of normal state samples mentioned above are taken as test samples. We use them to test the accuracy of the fault diagnosis model based on SVDD and D–S evidence theory. To compare the methods of the diagnosis model, we also calculate the accuracy of the method using only single state detection model and SLDM. The accuracy of the two methods in the simulation is shown in Table 9. The recognition accuracy of the proposed fault diagnosis model for normal state is 90%. The accuracy of locating fault circuit module is 93.08%. The effect of fault diagnosis model is better than that of SLDM.

The main reason is that when a single state detection model is used to identify circuit module faults, the performance of faults is different in time domain and frequency domain. The recognition rate of state detection model for different circuit faults is also uneven. Therefore, the SLDM has low accuracy and poor stability in fault location. The use of D–S evidence theory fully fuses the information of four state detection models. The new constructed evidence contains recognition information from different angles, and diagnostic results are more accurate and reliable. The validity of the proposed fault diagnosis model is proved by simulation experiments.

### 4.3. Hardware Experiment

**Figure 9:**
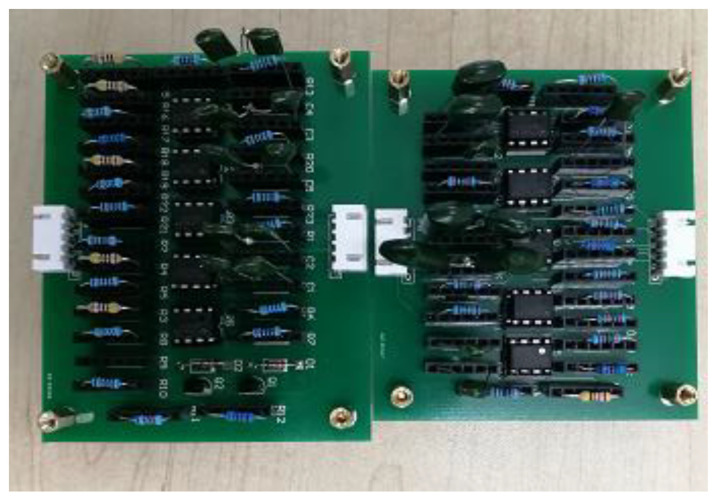
Experimental circuit board.

To verify the validity of the proposed method in practical application, we build a practical circuit for testing. The experimental circuit board is shown in Figure 9.

**Figure 10:**
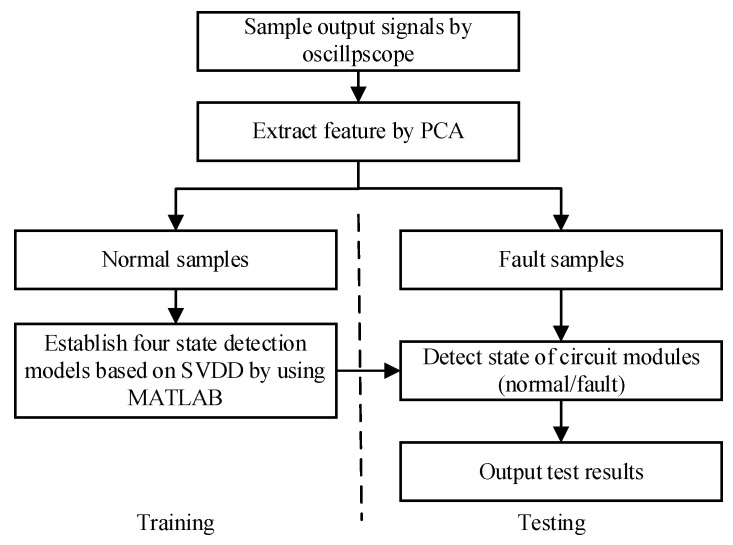
The hardware experimental process.

In the hardware experiment, the DC voltage source is used to supply the circuit with 15 V voltage, and the function signal generator is used to provide the excitation signals which are the same as simulation. In the process of data extraction, the upper computer and the oscilloscope are connected by the wire. Instrument communication is established through Agilent’s I/O equipment. BenchVue software is used to control the oscilloscope and sample the output signals of each module in normal and fault states. Pathon is used to process the data format, and MATLAB is used to verify the algorithm. The experiment proccess is shown as Figure 10.

In the verification of hardware experiment, the fault of circuit module is realized by plugging components with different parameters. The tolerance of capacitance resistance selected in the experiment is less than 5%. A total of 180 sets of test samples are obtained, including 150 sets of failure samples (30 sets per fault module) and 30 sets of normal state samples. The accuracy of the two methods in the experiment is shown in Table 10.

The table above shows that the recognition accuracy of the proposed fault diagnosis model for normal state is 80%. The accuracy of locating fault circuit module is 86.92%. The effect of fault diagnosis model is still better than that of SLDM. But the fault diagnosis rate of hardware experiment is slightly lower than that of simulation results. The main reason is the interference of ambient noise such as temperature and humidity in hardware experiment. The error of artificial measurement also affects the success rate of fault location. In addition, the D–S evidence body is generated by simulation results. Under actual conditions, component degradation and tolerance result in simulation data not fully reflecting the actual operation of the circuit. It makes the accuracy lower than simulation conditions. In summary, the simulation and hardware experiment prove the effectiveness of the proposed method in fault location of analog circuit modules. The method can locate fault module timely and accurately.

## 5. Conclusions

This paper proposes a fault diagnosis method to solve the problem of less negative samples and difficult fault location in analog circuit fault diagnosis. The Support Vector Data Description (SVDD) and Dempster–Schafer (D–S) evidence theory are applied to state detection and module fault location. The research results show that the proposed fault diagnosis method based on SVDD and D–S evidence theory can effectively solve the above problems. By using positive samples of the circuit, the method can accurately detect the operating state of circuit modules and determine which module is fault. A dual band-pass filter circuit is selected for simulation and hardware experiments, which fully verified the effectiveness of the proposed analog circuit fault diagnosis method. The research in this paper lays a certain foundation for the diagnosis of analog circuit faults by using single classification method. Future works should include the fault diagnosis for the states caused by the weak parameter drifts and multiple fault modules. Meanwhile, to improve the diagnosis accuracy, the parameter optimization method for the SVDD model will be also researched emphatically.

## Figures and Tables

**Table 1:** Dual band-pass circuit components and module function list.

Module	Components	Function
P1	R1 R2 RA RB C1 C2	High-pass filtering
P2	R7 R8 RG RH C7 C8	Low-pass filtering
P3	R5 R6 RE RF C5 C6	High-pass filtering
P4	R9 R10 R11	Adder
P5	R3 R4 RC RD C3 C4	Low-pass filtering

**Table 2:** Fault detection accuracy of TSG model (%).

Tset Point	Drift	P1 Fault	P2 Fault	P3 Fault	P4 Fault	P5 Fault
1	40%	**96.67**	7.61	7.56	7.56	8.00
	50%	**99.17**	7.50	7.83	7.89	10.13
2	40%	71.78	**86.39**	7.61	7.89	8.07
	50%	78.44	**92.56**	7.89	8.11	10.60
3	40%	30.83	6.17	**92.06**	5.89	5.87
	50%	35.22	6.11	**96.11**	6.22	7.73
4	40%	16.33	66.11	69.78	**98.11**	5.73
	50%	22.72	72.56	74.89	**99.89**	7.73
5	40%	11.33	37.44	73.22	81.22	**82.73**
	50%	12.44	44.72	75.67	87.56	**91.87**

**Table 3:** Fault average detection rate of TSG model (%).

Test Point	P1 Fault	P2 Fault	P3 Fault	P4 Fault	P5 Fault	Normal
1	**97.92**	7.55	7.70	7.73	9.07	92.33
2	75.11	**89.47**	7.75	8.00	9.34	92.33
3	33.03	6.14	**94.09**	6.06	6.80	93.67
4	19.52	69.34	72.34	**99.00**	6.73	94.33
5	11.89	41.08	74.45	84.39	**87.30**	93.33

**Table 4:** $m_{1}$ (TSG detection model).

Framework	P1 Fault	P2 Fault	P3 Fault	P4 Fault	P5 Fault	Normal
N1 abnormal	0.9792	0.0756	0.0770	0.0773	0.0907	0.0767
N2 abnormal	0.0105	0.7764	0.0042	0.0694	0.0791	0.0663
N3 abnormal	0.0017	0.0060	0.8011	0.0514	0.0561	0.0540
N4 abnormal	0.0007	0.0633	0.0364	0.7896	0.0517	0.0453
N5 abnormal	0.0003	0.0115	0.0104	0.0067	0.6257	0.0502
Normal	0.0025	0.0165	0.0036	0.0012	0.0910	0.7030
Uncertain	0.0052	0.0508	0.0673	0.0045	0.0058	0.0039

**Table 5:** $m_{2}$ (TSP detection model).

Framework	P1 Fault	P2 Fault	P3 Fault	P4 Fault	P5 Fault	Normal
N1 abnormal	0.7778	0.0295	0.0336	0.0317	0.0447	0.0333
N2 abnormal	0.0773	0.7515	0.0068	0.0290	0.0424	0.0275
N3 abnormal	0.0130	0.0094	0.7300	0.0500	0.0570	0.0500
N4 abnormal	0.0150	0.1051	0.1293	0.8438	0.0435	0.0355
N5 abnormal	0.0271	0.0201	0.0525	0.0326	0.6061	0.0682
Normal	0.0817	0.0426	0.0237	0.0113	0.2034	0.7840
Uncertain	0.0081	0.0419	0.0241	0.0016	0.0028	0.0014

**Table 6:** $m_{3}$ (FSG detection model).

Framework	P1 Fault	P2 Fault	P3 Fault	P4 Fault	P5 Fault	Normal
N1 abnormal	0.9792	0.0533	0.0533	0.0533	0.0533	0.0533
N2 abnormal	0.0045	0.8254	0.0034	0.0877	0.0877	0.0877
N3 abnormal	0.0010	0.0041	0.8211	0.0625	0.0625	0.0625
N4 abnormal	0.0001	0.0338	0.0232	0.7896	0.0290	0.0290
N5 abnormal	0.0001	0.0059	0.0048	0.0000	0.7181	0.0710
Normal	0.0001	0.0122	0.0029	0.0000	0.0425	0.6896
Uncertain	0.0150	0.0653	0.0912	0.0069	0.0069	0.0061

**Table 7:** $m_{4}$ (FSP detection model).

Framework	P1 Fault	P2 Fault	P3 Fault	P4 Fault	P5 Fault	Normal
N1 abnormal	0.4998	0.0367	0.0367	0.0367	0.0367	0.0367
N2 abnormal	0.0733	0.6591	0.0033	0.0310	0.0310	0.0310
N3 abnormal	0.1336	0.0094	0.8368	0.0310	0.0310	0.0310
N4 abnormal	0.0538	0.0071	0.0078	0.6301	0.0240	0.0240
N5 abnormal	0.0359	0.0095	0.0048	0.1646	0.6294	0.0263
Normal	0.1652	0.2555	0.0818	0.1055	0.2468	0.8499
Uncertain	0.0384	0.0227	0.0288	0.0011	0.0011	0.0010

**Table 8:** D–S evidence fusion results (Module P3 fault).

Evidence	$m_{1}$	$m_{2}$	$m_{3}$	$m_{4}$	$m_{\mathrm{final}}$
*m*(P1)	0.0770	0.0295	0.0533	0.0367	0.0238
*m*(P2)	0.0042	**0.7515**	0.0034	0.0310	0.0903
*m*(P3)	**0.8011**	0.0094	**0.8211**	0.0310	**0.6960**
*m*(P4)	0.0364	0.1051	0.0232	0.0240	0.0197
*m*(P5)	0.0104	0.0201	0.0048	0.0263	0.0034
*m*(normal)	0.0036	0.0426	0.0029	**0.8499**	0.1668
m(θ)	0.0673	0.0419	0.0912	0.0010	0.0000
Result	P3	P2	P3	Normal	**P3**

**Table 9:** Comparison between proposed method and SLDM (Simulation).

Circuit	State	SLDM				D–S
		TSG	TSP	FSG	FSP	
Dual band-pass	Normal	70%	78%	69%	86%	90%
filter circuit	Fault	79.33%	74.33%	83.00%	71.67%	93.00%

**Table 10:** Comparison between proposed method and SLDM (hardware experiment).

Circuit	State	SLDM				D–S
		TSG	TSP	FSG	FSP	
Dual band-pass	Normal	70%	65%	70%	75%	80%
filter circuit	Fault	76.92%	69.23%	82.69%	66.54%	86.92%

## Data Availability

Not applicable.
